# *BuT2* Is a Member of the Third Major Group of *hAT* Transposons and Is Involved in Horizontal Transfer Events in the Genus *Drosophila*

**DOI:** 10.1093/gbe/evu017

**Published:** 2014-01-22

**Authors:** Dirleane Ottonelli Rossato, Adriana Ludwig, Maríndia Deprá, Elgion L. S. Loreto, Alfredo Ruiz, Vera L. S. Valente

**Affiliations:** ^1^Programa de Pós-Graduação em Ecologia, Universidade Federal do Rio Grande do Sul (UFRGS), Porto Alegre, Rio Grande do Sul, Brazil; ^2^Laboratório de Genômica Funcional, Instituto Carlos Chagas (ICC), Fiocruz-PR, Curitiba, Paraná, Brazil; ^3^Programa de Pós-Graduação em Biologia Animal, Universidade Federal do Rio Grande do Sul (UFRGS), Porto Alegre, Rio Grande do Sul, Brazil; ^4^Departamento de Genética, Universidade Federal do Rio Grande do Sul (UFRGS), Porto Alegre, Rio Grande do Sul, Brazil; ^5^Programa de Pós-Graduação em Genética e Biologia Molecular Universidade Federal do Rio Grande do Sul (UFRGS), Porto Alegre, Rio Grande do Sul, Brazil; ^6^Departamento de Biologia, Universidade Federal de Santa Maria (UFSM), Santa Maria, Rio Grande do Sul, Brazil; ^7^Departament de Genètica i Microbiologia, Facultat de Biociènces, Universitat Autònoma de Barcelona, Spain

**Keywords:** *Drosophila*, transposase, *hAT*, MITE, horizontal transfer

## Abstract

The *hAT* superfamily comprises a large and diverse array of DNA transposons found in all supergroups of eukaryotes. Here we characterized the *Drosophila buzzatii BuT2* element and found that it harbors a five-exon gene encoding a 643-aa putatively functional transposase. A phylogeny built with 85 *hAT* transposases yielded, in addition to the two major groups already described, *Ac* and *Buster*, a third one comprising 20 sequences that includes *BuT2, Tip100*, *hAT-4_BM, and RP-hAT1.* This third group is here named *Tip*. In addition, we studied the phylogenetic distribution and evolution of *BuT2* by in silico searches and molecular approaches. Our data revealed *BuT2* was, most often, vertically transmitted during the evolution of genus *Drosophila* being lost independently in several species. Nevertheless, we propose the occurrence of three horizontal transfer events to explain its distribution and conservation among species. Another aspect of *BuT2* evolution and life cycle is the presence of short related sequences, which contain similar 5′ and 3′ regions, including the terminal inverted repeats. These sequences that can be considered as miniature inverted repeat transposable elements probably originated by internal deletion of complete copies and show evidences of recent mobilization.

## Introduction

Transposable elements (TEs) are widely distributed DNA sequences able to mobilize and increase their copy number within genomes. They are an important source of genetic variation in the genomes as a consequence of their insertion, domestication, and homologous recombination (Kidwell and Lisch 2001). Based on the transposition mechanism, via RNA or DNA intermediates, TEs can be classified into two major classes, retrotransposons (class I) and DNA transposons (class II), respectively (Finnegan 1989). These classes are further subdivided into subclass, order, superfamily, family, and subfamily, according to their sequence similarities and structural relationships (Wicker et al. 2007). Class II elements usually have terminal inverted repeats (TIRs) and encode a transposase that catalyzes their excision of the original site and promotes their reinsertion into a new place in the genome, generating target site duplications (TSDs; Wicker et al. 2007). The *hAT* superfamily comprise a large and diverse array of DNA transposons and related domesticated sequences found in all supergroups of eukaryotes including plants, animals, and fungi (Arensburger et al. 2011; Feschotte and Pritham 2007). Transposons of this superfamily are 2.5–5 kb in length, have relatively short TIRs (10–25 bp), and are flanked by 8-bp TSDs (Feschotte and Pritham 2007). Recently, the *hAT* superfamily was divided into two major groups or families, *Ac* and *Buster*, based on the primary sequence of their transposases and by differences in target-site selection (Arensburger et al. 2011). A small number of *hAT* transposons that do not fall into these two groups might comprise a third major group within the *hAT* superfamily (Arensburger et al. 2011; Zhang et al. 2013). 

Mobile elements are vertically transmitted through generations along with the rest of the genome. However, analyses of their distribution in different species showing inconsistencies between TE and species phylogenies suggest that horizontal transfer (HT) may take part of TE’s life cycle (Silva et al. 2004; Schaack et al. 2010; Wallau et al. 2012). Numerous cases of HT of TEs have been reported in *Drosophila,* involving elements from classes I and II, including members of the *hAT* superfamily, like the *hobo* element (Loreto et al. 2008).

Each TE class has both autonomous and nonautonomous elements. Autonomous elements have sequences encoding proteins needed for their transposition, whereas nonautonomous elements can be mobilized by enzymatic activities provided by autonomous elements. Miniature inverted repeat TEs (MITEs) encompass a particular group of class II nonautonomous elements. They are short sequences with no coding capacity and conserved TIRs that often reach high copy numbers in the genomes and are found within or near genes (Feschotte and Pritham 2007). They were first discovered in plants (Bureau and Wessler 1992) but are also found in several animal genomes, including *Drosophila* (Holyoake and Kidwell 2003; Ortiz et al. 2010; Dias and Carareto 2011; Deprá et al. 2012; Rius et al. 2013). The origin of some MITE families is unclear. Some of them seem to be derived from autonomous copies (Jiang et al. 2003, 2004; Zhang et al. 2004; Ortiz and Loreto 2008; Deprá et al. 2012) although others are apparently the result of recombination events producing a pair of TIRs that are equal or similar to those of an autonomous element that will provide the transposase for MITE mobilization (Jiang et al. 2004).

In the genus *Drosophila*, TE insertions have often been found in the breakpoints of chromosomal inversion (Lim 1988; Lyttle and Haymer 1992; Eggleston et al. 1996; Regner et al. 1996; Evgen’ev et al. 2000; Cáceres et al. 2001). Cáceres et al. (2001) characterized the breakpoints of a *Drosophila buzzatii* polymorphic inversion, which have accumulated insertions of several different TEs. One of them, called *BuT2*, was tentatively classified in the *hAT* superfamily of class II transposons. This element is relatively scarce in the *D. buzzatii* genome (Casals et al. 2006), but its presence in the inversion breakpoints indicates recent transpositional activity. In this work, we seek to characterize the *BuT2* element and contribute to the knowledge of *hAT* superfamily evolution. We found that *BuT2* harbors a five-exon gene encoding a 643-aa transposase and phylogenetically classify it in the third major group of *hAT* transposons that we named the *Tip* family. By in silico searches in genomes and molecular biology approaches, we conducted a screening covering 105 insect species, of which 72 belong to the genus *Drosophila.* Our results show *BuT2* sequences are present in five *Drosophila* groups and were horizontally transmitted between some of them. We also found in some species short nonautonomous sequences related to *BuT2*. These sequences have conserved TIRs and probably originated by deletion of *BuT2* autonomous copies and may represent the rising of a MITE family.

## Materials and Methods

### In Silico Searches on Insect Genomes

We investigated the presence of *BuT2* homologous sequences in 21 sequenced *Drosophila* genomes and in 27 other insect genomes (table 1). These genomes are deposited in the FlyBase database (http://flybase.bio.indiana.edu/blast/, last accessed February 4, 2014; Grumbling and Strelets 2006). *Drosophila buzzatii* canonical *BuT2* nucleotide sequence (GenBank AF368884) was used as query on BlastN and TBlastX. We used an e*-*value cutoff of 1e-20. To calculate the average similarity between *BuT2* and the sequences found*,* the similarity information of all high-scoring segment pairs (HSPs) from the significant hits were used.

**Table 1:** Number of Significant Hits Found Using BlastN and TBlastX Tools in Flybase and the Percent Average Similarity Found with the Query

**Species**	**BlastN**		**TBlastX**	
	Hits	Average Similarity	Hits	Average Similarity
***D. melanogaster***	0	–	0	–
***D. simulans***	0	–	0	–
***D. sechellia***	0	–	0	–
***D. yakuba***	0	–	8	53.16
***D. erecta***	0	–	0	–
***D. ficusphila***	3	81.90	26	52.81
***D. eugracilis***	1	82.63	10	55.03
***D. biarmipes***	0	–	11	51.21
***D. takahashii***	0	–	7	47.33
***D. elegans***	0	–	5	50.75
***D. rhopaloa***	0	–	16	51.30
***D. kikkawai***	2	81.83	29	54.94
***D. ananassae***	0	–	10	49.91
***D. bipectinata***	1	82.60	46	52.86
***D. pseudoobscura***	0	–	6	48.76
***D. persimilis***	0	–	10	49.17
***D. miranda***	0	–	2	48.99
***D. willistoni***	28	87.48	55	65.50
***D. mojavensis***	3	90.17	28	56.54
***D. virilis***	0	–	0	–
***D. grimshawi***	0	–	0	–
***Culex quinquefasciatus***	0	–	0	–
***A. aegypti***	0	–	8	38.54
***An. gambiae***	0	–	1	41.82
***Mayetiola destructor***	0	–	0	–
***B. mori***	0	–	0	–
***Danaus plexippus***	0	–	1	41.96
***T. castaneum***	0	–	32	42.47
***N. giraulti***	0	–	11	42.23
***N. longicornis***	0	–	15	41.43
***N. vitripennis***	0	–	27	41.6
***Apis mellifera***	0	–	0	–
***Apis florea***	0	–	0	–
***Bombus impatiens***	0	–	0	–
***Bombus terrestris***	0	–	0	–
***Megachile rotundata***	0	–	5	39.27
***Acromyrmex echinatior***	0	–	9	45.06
***Atta cephalotes***	0	–	1	38.74
***C. floridanus***	0	–	7	38.93
***Harpegnathos saltator***	0	–	10	37.68
***Linepithema humile***	0	–	24	38.88
***Pogonomyrmex barbatus***	0	–	2	46.91
***Solenopsis invicta***	0	–	83	43.16
***A. pisum***	0	–	59	43.43
***R. prolixus***	0	–	0	–
***Pediculus humanus corporis***	0	–	0	–
***Ixodes scapularis***	0	–	54	43.22
***Rhipicephalus microplus***	0	–	0	–

Note.–The query sequence was the canonical *But2* sequence from *D. buzzatii*.

The presence of short sequences related to *BuT2* was investigated by in silico polymerase chain reaction (PCR) using the BlastN tool against all 21 *Drosophila* genomes. The query was a sequence formed by the BuT2_F primer followed by the reverse complementary sequence of BuT2_R primer. These primers are described below. Hits that visibly contained both regions of these primers, which correspond in part to the *BuT2* TIRs, were analyzed looking for conserved TIRs and TSDs.

The identity of sequences and some insertions found in *BuT2* copies was investigated by Blast tool against the GenBank (Altschul et al. 1990) or using CENSOR (Kohany et al. 2006), a software tool that screens query sequences against the Repbase Update (Jurka et al. 2005), a database of repetitive sequences eukaryotes.

### Fly Stocks and DNA Manipulation

Flies are maintained in laboratory by mass crosses and cultivated in corn flour culture medium in a constant temperature chamber (20 °C). Genomic DNA was extracted from adult flies as described (Sassi et al. 2005). A total of 67 species (table 2) belonging to genus *Drosophila*, *Zaprionus,* and *Scaptodrosophila* were used in the laboratory experimental approaches. One strain of each species was used, and their origin information is available in the supplementary table S1, Supplementary Material online.

**Table 2:** Drosophilidae Species Investigated by PCR, Dot Blot, and BlastN Approaches, with Their Taxonomic Placement and Respective Results

Genus	Subgenus	Group	Species	PCR		Dot	BlastN
				1	2		
***Drosophila***	*Drosophila*	*guarani*	*D. ornatifrons*	−	−	w	na
			*D. subbadia*	−	−	w	na
			*D. guaru*	−	−	w	na
		*grimshawi*	*D. grimshawi*	−	−	na	−
		*guaramuru*	*D. griseolineata*	−	−	−	na
			*D. maculifrons*	−	−	−	na
		*tripunctata*	*D. nappae*	−	−	w	na
			*D. paraguayensis*	−	−	na	na
			*D. crocina*	−	−	−	na
			*D. paramediostriata*	−	−	−	na
			*D. tripunctata*	−	−	−	na
			*D. mediodiffusa*	−	−	−	na
			*D. mediopictoides*	−	−	−	na
		*cardini*	*D. cardini*	−	−	na	na
			*D. cardinoides*	−	−	−	na
			*D. neocardini*	−	−	−	na
			*D. polymorpha*	−	−	−	na
			*D. procardinoides*	−	−	−	na
			*D. arawakana*	−	−	−	na
		*pallidipennis*	*D. pallidipennis*	+	+	+	na
		*calloptera*	*D. ornatipennis*	−	−	w	na
		*immigrans*	*D. immigrans*	−	−	−	na
		*funebris*	*D. funebris*	−	−	−	na
		*mesophragmatica*	*D. gasici*	−	−	−	na
			*D. brncici*	−	−	−	na
			*D. gaucha*	−	−	−	na
			*D. pavani*	−	−	−	na
		*repleta*	*D. hydei*	−	−	−	na
			*D. mojavensis*	−	−	+	+
			*D. buzzatii*	+	+	+	na
			*D. mercatorum*	−	−	+	na
			*D. repleta*	−	−	na	na
		*canalinea*	*D. canalinea*	−	−	na	na
		*flavopilosa*	*D. cestri*	−	−	na	na
			*D. incompta*	−	−	+	na
		*virilis*	*D. virilis*	−	−	−	−
		*robusta*	*D. robusta*	−	−	−	na
	*Sophophora*	*melanogaster*	*D. melanogaster*	−	−	−	−
			*D. simulans*	−	−	−	−
			*D. sechellia*	−	−	na	−
			*D. mauritiana*	−	−	−	na
			*D. teissieri*	−	−	−	na
			*D. santomea*	−	−	−	na
			*D. erecta*	−	−	−	−
			*D. yakuba*	−	−	−	−
			*D. kikkawai*	−	−	w	+
			*D. ananassae*	−	−	−	−
			*D. malerkotliana*	−	−	w	na
			*D. orena*	−	−	−	na
			*D. ficusphila*	na	na	na	+
			*D. eugracilis*	na	na	na	+
			*D. biarmipes*	na	na	na	−
			*D. takahashii*	na	na	na	−
			*D. elegans*	na	na	na	−
			*D. rhopaloa*	na	na	na	−
			*D. bipectinata*	na	na	na	+
		*obscura*	*D. pseudoobscura*	−	−	−	−
			*D. persimilis*	na	na	na	−
			*D. miranda*	na	na	na	−
		*saltans*	*D. prosaltans*	−	+	+	na
			*D. saltans*	−	+	+	na
			*D. neoelliptica*	−	−	w	na
			*D. sturtevanti*	−	+	w	na
		*willistoni*	*D. sucinea*	+	−	+	na
			*D. nebulosa*	+	−	+	na
			*D. paulistorum*	+	−	+	na
			*D. willistoni*	+	+	+	+
			*D. equinoxialis*	+	−	+	na
			*D. insularis*	−	−	−	na
			*D. tropicalis*	−	−	−	na
			*D. capricorni*	+	−	+	na
	*Dorsilopha *		*D. busckii*	−	−	−	na
***Zaprionus***			*Z. indianus*	−	−	−	na
			*Z. tuberculatus*	−	−	−	na
			*Z. sepsoide*	−	−	na	na
***Scaptodrosophila***			*S. latifasciaeformis*	−	−		na
			*S. lebanonensis*	−	−		na

Note.— −, no amplification, hybridization signal, or significant hit on BlastN obtained; +, positive amplification, hybridization signal, or significant hit on BlastN; w, weak signal in the dot blot; na, not available/analyzed.

### Dot Blot

We used dot blot to investigate the presence of *BuT2* in 60 Drosophilidae species (table 2). Approximately 1 μg of genomic DNA, denatured by heat, was applied directly on a nylon membrane (Hybond-N+, GE Healthcare). Hybridization and detection followed the protocol of the kit CPD-Star Detection Module (GE Healthcare). The PCR fragment amplified from a *D. willistoni BuT2* clone (Bf2_Dwil1) was used as probe and was labeled with the Gene Images Kit AlkPhos Direct Labelling Module (GE Healthcare). The hybridization temperature was 55 °C.

### PCR Screening

**Fig. 1.—:**
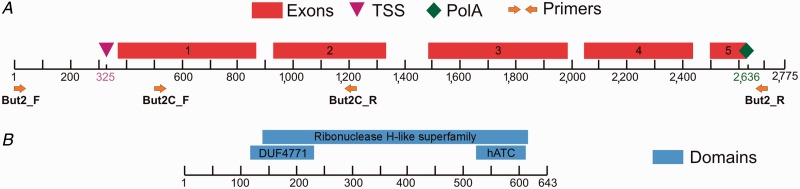
Schematic representation of *BuT2* nucleotide and predicted protein. *A*: Organization of *D. buzzatii BuT2* coding sequences, TSS, transcription start site; Exons 1–5; PolA, polyadenylation signal. Arrows indicate the primer annealing regions. *B*: Organization of *D. buzzatii BuT2* predicted amino acid sequence with the domains found.

PCR approach was also used to investigate the presence of *BuT2* in 67 Drosophilidae species (table 2). Four different primers were designed (fig. 1*A*). Primers BuT2_F 5′ CAGTGCTGCCAACAWTTYGT 3′ and BuT2_R 5′ CASTGCTGCCAATTTAGCYA 3′ were designed based on three sequences: the canonical *BuT2* element from *D. buzzatii* (AF368884.1), the *BuT2* sequence located in the scf2_1100000004958:2664879-26680344 (scf1_Dwil) of *D. willistoni* genome, and the one located in the scaffold_3367: 3535-7555 (scf1_Dmoj) of *D. mojavensis* genome. These primers were designed to amplify the complete *BuT2* element and are degenerated in some positions. Two other primers were designed based on the same sequences cited above from *D. buzzatii* and *D. willistoni*. The nucleotide sequences are: BuT2C_F 5′ AGACYTCGGGRACAGTTTTGC 3′ and BuT2C_R 5′ AGCATTAATGCYAARCTTTC 3′. The following protocol for the PCR reactions was used: 50 ng of genomic DNA added to a solution of 2.5 mM MgCl_2_, 1× buffer reaction, 200 mM of each deoxynucleotide, 20 pmol of each primer, and 1 U of Taq polymerase in 50 µl of total volume. The condition of reactions were 96 °C for 2 min, followed by 30 cycles of 96 °C for 30 s, 55 °C for 45 s, and 72 °C for 1–3 min, depending on the expected size of the fragment. PCR products were cloned using TOPO TA cloning system (Invitrogen) and selected clones were sequenced from PCR products purified with Exonuclease I (USB) and Shrimp Alkaline Phosphatase (USB) on MegaBACE 500 automated sequencer or by a sequencing service (www.macrogen.com, last accessed February 7, 2014).

### Searching for a Transposase Coding Region within *BuT2*

To check whether the complete copies of the *BuT2* potentially encodes for a functional transposase, we used three programs to predict the existence of possible introns and coding regions: GeneMark.hmm (Lomsadze et al. 2005), GENSCAN (Burge and Karlin 1997), and FGENESH (Yao et al. 2005). The Simple Modular Architecture Research Tool (SMART) (Letunic et al. 2009, 2012) and InterProScan (Quevillon et al. 2005) tools were used to check for domains in the predicted protein sequences.

### Sequence Analysis

Nucleotide sequences were aligned using Muscle (Edgar 2004) and *BuT2* phylogeny was inferred by three methods: Neighbor-Joining (NJ) and maximum likelihood (ML) using the Tamura 3-parameter substitution model (Tamura 1992) with gamma parameter equaling 3.0 as indicated by model selection analysis and Bayesian analysis (BA) with parameters set to nst = 2 using a gamma distribution. NJ and ML trees were implemented in Mega5.2 (Tamura et al. 2011), and 1,000 replicates bootstrap was used to access the reliability of branches. BA was implemented in MrBayes 3.1.2 (Ronquist et al. 2012) with at least 1,000,000 generations and a burn-in of 25%.

We also investigated the phylogenetic placement of *BuT2* transposase using the transposase amino acid sequences from several *hAT* superfamily members collected based on Arensburger et al. (2011). We also carried out BlastP searches using as query the predicted *BuT2* transposase amino acid sequence against all nonredundant protein sequences. We retrieved all sequences with a minimum identity of 30% and minimum coverage of 60% with an e*-*value cutoff of 5e-20. The accession numbers of these sequences are listed in supplementary table S2, Supplementary Material online. Protein sequences were aligned using M-Coffee, which computes a consensus alignment from several multiple sequence alignment programs (Moretti et al. 2007). Conserved regions in the alignment were selected to infer the transposase phylogeny. We performed ML and NJ using the rtREV model (Dimmic et al. 2002) + G + F, as indicated by model selection implemented on Mega5.2 (Tamura et al. 2011).

Sequences of two genes *alpha methyl dopa* (*Amd*) and *alcohol dehydrogenase* (*Adh*) were used to compare their divergence with those found for *BuT2* sequences with the purpose of testing the HT hypothesis. P-distance between sequences was calculated for *BuT2* and for the nuclear genes using Mega5.2 (Tamura et al. 2011). *Adh* and *Amd* genes sequences were obtained from GenBank or by BlastN against the genomes. Accession numbers or scaffold coordinates are given in supplementary table S3, Supplementary Material online. A χ^2^ test was used to verify whether the divergence observed for *BuT2* between species is significantly different from the expected divergence based on nuclear genes *Adh* or *Amd*. Vertical transmission (VT) can be assumed if the *BuT2* divergence is greater or equal than those from the nuclear genes. On the other hand, if the *BuT2* divergence is smaller than the nuclear gene divergence, HT can be suggested. Similar approach was already used to investigate HT events (Ludwig and Loreto 2007).

## Results

### *BuT2* from *D. buzzatii* Encodes a Putatively Functional Transposase

A single copy of the *D. buzzatii* transposon *BuT2* has been described (Cáceres et al. 2001). It is 2,775-bp long, possesses 12-bp TIRs, and is flanked by 8-bp TSDs. We searched this copy for sequences encoding the transposase using three de novo gene predictors. FGENESH software predicted a transcription start site (TSS) at position 325, five exons (nucleotide positions: 366–864; 925–1331; 1486–1985; 2044–2436; 2495–2627) encoding a 643-aa protein and a polyadenylation signal at position 2636 (fig. 1*A*). Similarly, GeneMark.hmm and GENSCAN predicted five-exon genes but encoding somewhat shorter proteins (599 and 520-aa, respectively). We choose FGENESH as the best prediction because the protein is longer and similar in size to many other transposases of active *hAT* transposons, for instance, those of *hobo* element in the fruit fly *D. melanogaster* (658 aa; Calvi et al. 1991), *Hermes* in *Musca domestica* (612 aa; Warren et al. 1994), or *TcBuster* in *Tribolium castaneum* (636 aa; Arensburger et al. 2011). In addition, bioinformatic and phylogenetic observations (see later) support that this is likely the correct *BuT2* transposase.

We used two different computer programs to search for domains within the 643-aa *BuT2* protein (fig. 1*B*). SMART showed the presence of a hATC domain in residues 515–603 (e-value = 2.8e-06), which is a highly conserved dimerization domain (pfam05699) found in DNA transposons from the *hAT* superfamily (Essers et al. 2000). InterProScan found, in addition to the *hAT* dimerization domain, a domain of unknown function DUF4371 in residues 116–229 (e-value = 1.8e-8) and a Ribonuclease H-like superfamily domain (SSF53098) in residues 138–608 (e-value = 4.4e-24). This is a structural domain consisting of a three-layer alpha/beta/alpha fold that contains mixed beta sheets and is found in some ribonucleases, retroviral integrases, transposases, and exonuclease, suggesting they share a similar mechanism of catalysis (Gough et al. 2001). We conclude *D. buzzatii BuT2* encodes a putatively functional transposase related to those of the *hAT* superfamily.

### *BuT2* Belongs to the Third Major Group of *hAT* Transposons

**Fig. 2.—:**
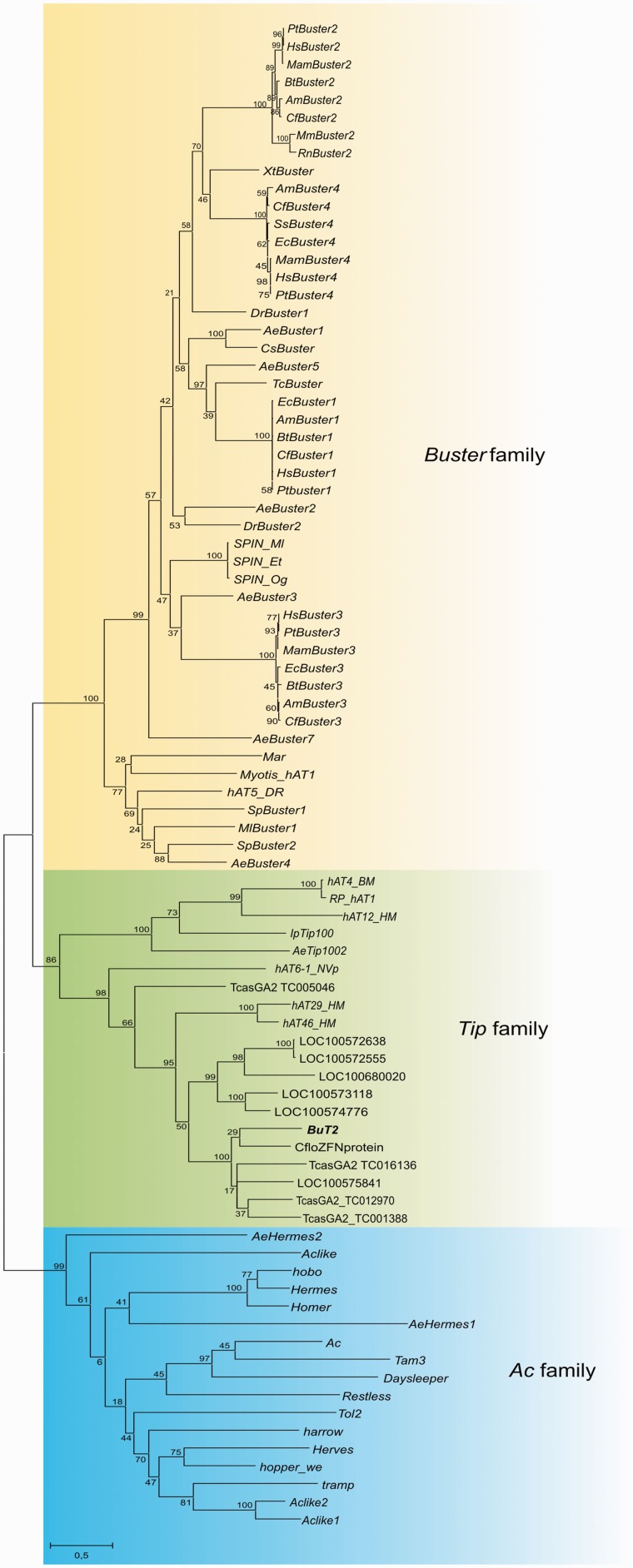
Unrooted ML phylogenetic tree of *hAT* elements amino acid transposase sequences. Node supports are bootstrap values (1,000 replications). The three proposed *hAT* families, *Ac*, *Buster,* and *Tip* are shown.

The *hAT* superfamily comprises a complex array of transposons found in diverse eukaryotic supergroups (Feschotte and Pritham 2007; Arensburger et al. 2011). Two main groups, named *Buster* and *Ac*, were established by Arensburger et al. (2011). Recently, Zhang et al. (2013) described a novel *hAT* element horizontally transmitted between *Bombyx mori* (*hAT-4_BM*) and *Rhodnius prolixus* (*RP-hAT1*), which might represent a third group well separated from the previous ones, *Buster* and *Ac*. In order to establish the relationships of *BuT2* with the other members of the *hAT* superfamily, we built a phylogeny with the transposase amino acid sequences described previously (Arensburger et al. 2011; Zhang et al. 2013) along with other 14 homologous sequences (supplementary table S2, Supplementary Material online). These new sequences correspond to three proteins annotated in Repbase as belonging to *hAT* transposons (*hAT-29_HM* and *hAT-46_HM* from *Hydra magnipapillata* and *hAT6-1_NV*p from *Nasonia vitripennis*) and four proteins of *T. castaneum*, five proteins of *Acyrthosiphon pisum,* and one protein of *Camponotus floridanus* and *N. vitripennis* retrieved from Protein databases by a BlastP search*.* None of the latter sequences has been annotated as a transposase, although all of them contain the hATC dimerization domain. The phylogenetic tree revealed three major clades with many members in each clade (fig. 2). Two of them correspond to the known groups *Ac* and *Buster*, whereas the third clade comprises 20 proteins including the transposases of *BuT2, Tip100*, *hAT-4_BM, and RP-hAT1.* This third major group has been here named as the *Tip* group after the transposon *Tip100* from the common morning glory *Ipomoea purpurea* (Habu et al. 1998)*. BuT2* is the only transposon from the *Tip* group known in *Drosophila*.

### In Silico Searches Reveal the Presence of *BuT2* in the *melanogaster, repleta, and willistoni* Species Groups

We searched for sequences similar to *BuT2* in the genomes of 21 *Drosophila* species and 27 other insects available in FlyBase (table 1) using BlastN. Significant hits (number in parentheses) were found in *D. ficusphila* (3)*, D. eugracilis* (1)*, D. kikkawai* (2)*, D. bipectinata* (1)*, D. mojavensis* (3), and *D. willistoni* (28). Information about the length and scaffold position of these sequences is given in supplementary table S4, Supplementary Material online. Sequences similar to *BuT2* in the four species of the *melanogaster* group (*D. ficusphila, D. eugracilis, D. kikkawai, D. bipectinata*) are relatively short (165–836 bp) with identity ∼80%. Furthermore, none of these sequences seems to include TIRs nor is flanked by TSDs.

In *D. mojavensis*, which belongs to the *repleta* group of *Drosophila* subgenus as *D. buzzatii*, we found only three significant hits (supplementary table S4, Supplementary Material online). The most complete copy is 4,017-bp long, has 12-bp TIRs (with two mismatches), and is flanked by identical 8-bp TSDs. This copy is 91.2% identical to *D. buzzatii BuT2* copy but has a deletion of 392 bp and an insertion of approximately 1,700 bp (likely a *mariner* element as identified by CENSOR). The other two copies in *D. mojavensis* are incomplete and have, respectively, 4,928 bp and 1,128 bp. The large size of the 4,928-bp copy is due to one large insertion (∼2,600 bp).

In *D. willistoni*, a species belonging to the subgenus *Sophophora*, we retrieved 28 significant hits, but only two copies appear to have large segments of the transposase (supplementary table S4, Supplementary Material online). The remaining copies were smaller (800–1,000 bp) and seemed to lack the internal portion of the element (coding for the transposase) but conserve the outermost portions (that are presumably required for transposition). Therefore, there seem to be nonautonomous copies generated by deletion (see later). The most complete copy has 3,156 bp length including 12-bp TIRs and is flanked by 8-bp TSDs (with one mismatch). This copy is 92.4% identical to *D. buzzatii BuT2* and harbors a similar five-exon gene with conserved splice sites and encoding a 642-aa protein that is 90% identical to that of *D. buzzatii* (after correction of a mutation in the first exon that generates a stop codon). This *D. willistoni* copy is longer than that of *D. buzzatii* because it possesses within intron 2 an insertion of 424 bp (seemingly a *BEL* LTR retrotransposon as identified by CENSOR). The other copy has 6,093-bp length, including a deletion of 355 bp and an insertion of approximately 4,200 bp (a *Minos* transposon according to CENSOR). The *BuT2* most complete copies of *D. buzzatii*, *D. mojavensis*, and *D. willistoni* show, after removal of secondary TE insertions, an unexpected identity (>90%) raising the hypothesis of HT among the species (see later).

Additional bioinformatic searches were carried out using TBlastX, a more sensitive search, because it uses translated DNA queries and subjects and compares the resulting amino acid translations. Significant hits were recovered, in addition to the species already known to harbor *BuT2* sequences, in *D. yakuba*, *D. biarmipes*, *D. takahashii, D. elegans, D. rhopaloa, D. ananassae, D. pseudoobscura, D. persimilis, and D. miranda.* Significant hits were also found in 17 other insect genomes (table 1). However, all these sequences present low identity with *BuT2* element (in general, less than 50% of amino acid identity), suggesting these sequences may correspond to other *hAT* elements rather than *BuT2*.

### Experimental Searches Show a Patchy Distribution of *BuT2* among Drosophilid Species

We used dot blot hybridization to test for the presence of sequences similar to *BuT2* in 60 Drosophilid species (table 2). Results are shown on supplementary figure S1, Supplementary Material online. Dot blot filters showed strong hybridization signals in three species known to harbor *BuT2* (see above) and included as positive controls: *D. buzzatii*, *D. mojavensis,* and *D. willistoni*. In contrast, no hybridization signals were found in four species known to lack *BuT2* (see above) and included here as negative controls: *D. virilis*, *D. melanogaster*, *D. erecta,* and *D. simulans*. In addition, we observed strong hybridization signals in all species of the sister groups *willistoni* and *saltans*, as well as in *D. pallidipennis* (*pallidipenis* species group) and *D. incompta* (*flavopilosa* species group). Other species showed a weak signal, including *D. kikkawai,* that presented a sequence similar to *BuT2* by in silico searches*.*

We also used two PCR assays in 67 Drosophilid species to investigate the distribution of *BuT2* (table 2). In PCR 1, we used primers BuT2_F and BuT2_R, expected to amplify the complete *BuT2* element (∼2,770 bp), as shown in figure 1*A*. The amplified fragments with these primers were much smaller than expected and had a variable size among species, even within the same species group. None of the species showed an amplification corresponding to a complete element. These small fragments were cloned and sequenced, and all of them correspond to small sequences related to *BuT2.* Possibly, due to their smaller size and perhaps larger frequency in the genomes, these fragments are amplified with preference to the complete element, if present. The presence of small sequences related to *BuT2* was confirmed by in silico PCR in the *D. willistoni* genome that recovered 24 short sequences, with size ranging from 532 to 927 bp with an average (±SD) = 739 bp (±108). Most (18) of these sequences present TIRs highly similar to those of the complete *BuT2* copy and 14 are flanked by identical TSDs (supplementary table S5, Supplementary Material online). For instance, the copy in scaffold_4830 (Scf13_Dwil) is 773-bp long and is 96.8% identical to *BuT2* in the first 94 nt and 95.3% identical in the terminal 128 nt. The high similarity in the outermost sequences, including TIRs, is very significant, because these sequences are presumably required for transposition.

Looking for complete *BuT2* copies, the same collection of species was screened by PCR 2, with additional primers BuT2C_F and BuT2C_R covering a 750-bp central region of element *BuT2* (fig. 1). Table 2 shows the PCR results for both fragments. Amplicons were cloned and sequenced for the following species: Fragment 1, *D. pallidipennis*, *D. buzzatii*, *D. sucinea*, *D. nebulosa*, *D. paulistorum*, *D. capricorni*, *D. equinoxialis*; Fragment 2: *D. willistoni*, *D. buzzatii* and *D. pallidipennis*, *D. prosaltans*, *D. saltans*, *D. sturtevanti* and *D. willistoni*. The GenBank accession numbers of these are shown in supplementary table S6, Supplementary Material online.

Our data show that *BuT2* has a patchy distribution among *Drosophila* species being found in species from five species groups: *pallidipennis* and *repleta* of subgenus *Drosophila* and *melanogaster, saltans,* and *willistoni* from subgenus *Sophophora.* Nevertheless, we cannot discard the possibility of *BuT2* presence in some other group not tested in this work, and divergences in the primer regions may have led to a negative PCR result for some species harboring *BuT2*. It could have happened to *D. incompta, D. insularis,* and *D. tropicalis*, which showed a relatively strong hybridization signal in dot blot.

### *BuT2* Phylogenetic and Divergence Analyses

**Fig. 3.—:**
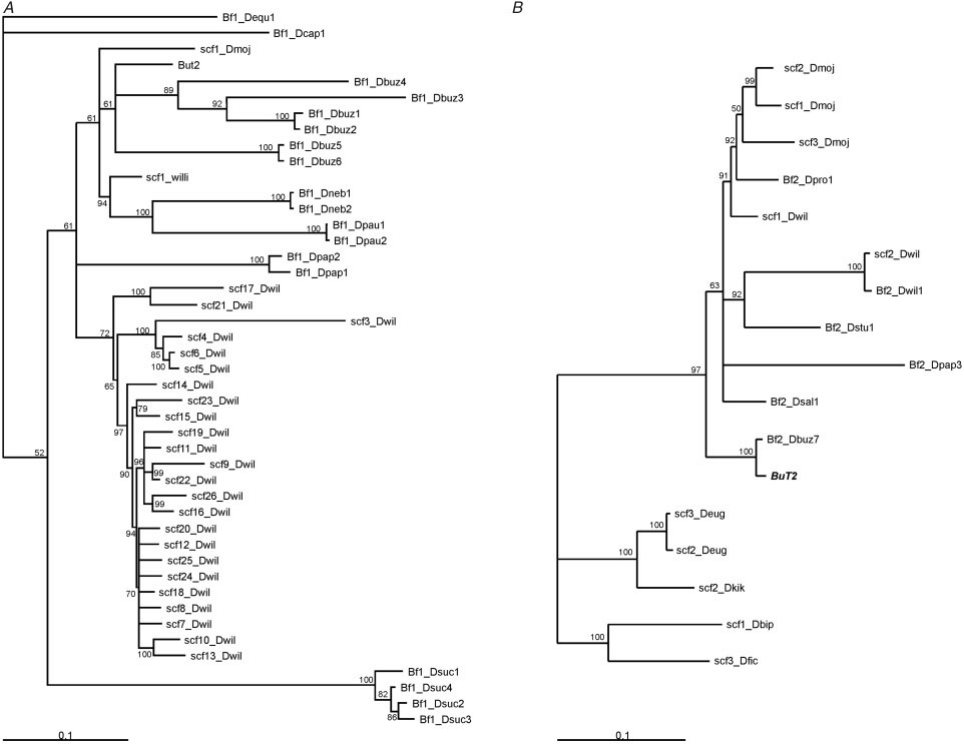
Phylogenetic relationships of *BuT2* copies and associated MITE sequences*. A*: BA of the *BuT2* short sequences obtained using the BuT2_F and BuT2_R primers (Bf1) and by in silico PCR (scf). *B*: BA of *BuT2* copies obtained with the primers BuT2C_F and BuT2CR (Bf2) and by in silico searches (scf). Node supports are shown by posterior probability (only above 50%). *Drosophila buzzatii BuT2* canonical sequence is shown in boldface. The species are the following: Dequ, *D. equinoxialis*; Dcap, *D. capricorni*; Dmoj, D. mojavensis; Dbuz, *D. buzzatii*; Dwil, *D. willistoni*; Dneb, *D. nebulosa*; Dpau, *D. paulistorum*; Dpap, *D. pallidipennis*; Dsuc, *D. sucinea*; Dfic, *D. ficusphila*; Dbip, *D. bipectinata*; Dsal, *D. saltans*; Dstu, *D. sturtevanti*; Dpro, *D. prosaltans*; Dkik, *D. kikkawai*; Deug, *D. eugracilis*.

All amplicons generated with primers BuT2_F and BuT2_R can be considered as MITEs because they contain only the boundaries of *BuT2* element, including TIRs. These sequences together with those obtained by in silico PCR and the corresponding homologous region of complete elements from *D. buzzatii*, *D. willistoni,* and *D. mojavensis* were used to construct a phylogeny (fig. 3*A*). The phylogenetic methods applied, NJ, MP, and BA, showed similar trees with low support of branches, which hampers the interpretation of relationships among species. When nodes with bootstrap or posterior probabilities values below 50% were forced to collapse, the phylogeny became almost an entire polytomic tree (not shown). The only well-established relationships are some species-specific clades grouping the sequences of *D. sucinea*, *D. buzzatii*, *D. willistoni,* and a clade grouping together sequences of *D. willistoni*, *D. nebulosa,* and *D. paulistorum*. The 24 MITE sequences from *D. willistoni* are grouped in the tree in a single clade somewhat separated from the complete copy and the short length of some branches indicates those copies are very similar, suggesting recent amplification.

Sequences of fragments amplified with BuT2C_F and BuT2C_R were also used to infer a *BuT2* phylogeny (fig. 3*B*). NJ, MP, and BA phylogenies also showed similar trees, and the relationship between several species is unclear, because bootstrap and posterior probabilities values are very low for some branches. We can observe a confident clade grouping *D. prosaltans* and *D. mojavensis* and another one containing *D. sturtevanti* and *D. willistoni.* Both clades clustered with sequences from *D. pallidipennis*, *D. saltans,* and *D. buzzatii BuT2*. Another clade contains sequences of *D. kikkawai* and *D. eugracilis*.

Both phylogenies present low resolution in several nodes, and it may be inherent of this transposon sequence if relationships may not be demonstrated by simple branch bifurcations in the trees. It can represent multiple/simultaneous divergence events (Maddison 1989).

**Fig. 4.—:**
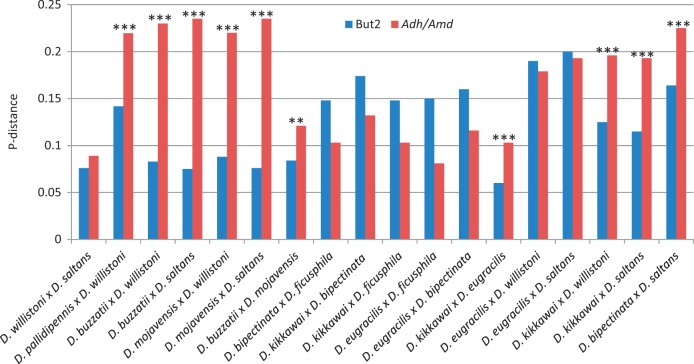
Comparative analysis of the divergence found among the *BuT2* sequences and the nuclear genes *Adh* and *Amd*. A χ^2^ test was used to verify whether the *BuT2* observed divergence is significantly different from the expected based on the *Adh* and *Amd* genes. Results of χ^2^ test: ***P < 0.001; **P < 0.01.

In order to test HT hypothesis, we compared the interspecific divergence found among the *BuT2* sequences, with the divergence of the nuclear genes *Adh* and *Amd*. Given the functional relevance of these genes, it is expected that they are under high selective constraints. Thus, theoretically, HT events can be inferred when the divergence of *BuT2* is significantly lower to the divergence found for these genes. Pairwise divergences for *BuT2* and genes were estimated for all species, and the most relevant comparisons are shown in figure 4.

All comparisons between species from *saltans* group and *D. willistoni* show, for *BuT2,* similar or greater divergence than those for the genes. In figure 4 is exemplified the comparison of *D. willistoni* and *D. saltans,* showing nonsignificant difference for *BuT2* and *Adh* divergence, corroborating our hypothesis of the *BuT2* presence in the ancestor of *willistoni* and *saltans* groups followed by VT during speciation processes.

*Drosophila pallidipennis* has no *Adh* sequence available*,* then we used the *Amd* gene, and the comparison with *D. willistoni* shows much smaller divergence for *BuT2* than the expected based on *Amd* divergence, suggesting HT.

*BuT2* sequences from *D. buzzatii* and *D. mojavensis* are much more similar to all those from *willistoni* and *saltans* species than would be expected for VT based on *Adh* gene (fig. 4). We also compared *D. buzzatii* with *D. mojavensis* (*Adh* gene), and these comparisons also show *BuT2* divergences are significantly lower than the gene divergence.

The relationships of *BuT2* sequences are more complex to understand when we consider the *melanogaster* group where these sequences are present in four species with a scattered distribution and phylogenetic inconsistencies. The results of *BuT2* and *Adh* divergence pairwise comparisons among *D. ficusphila*, *D. bipectinata, D. kikkawai,* and *D. eugracilis* indicate VT, except for *D. kikkawai* and *D. eugracilis* that indicates HT. Comparisons among these species with *D. willistoni* and *D. saltans* indicate VT for most of comparisons (comparisons of *D. eugracilis* with *D. willistoni* and *D. saltans* are exemplified in fig. 4). HT was suggested for the comparisons of *D. willistoni* and *D. saltans* with *D. kikkawai* and for *D. bipectinata* with *D. saltans*; however, *BuT2* sequences of these two species are very short and the results may be probably biased.

## Discussion

### *Drosophila buzzatii BuT2* Encodes a Putatively Functional Transposase and Belongs to the Third Major Group of *hAT* Transposons

Our results from gene prediction programs suggest that the *BuT2* copy of *D. buzzatii* encodes a putatively active transposase, which is in agreement with the recent transpositional activity inferred in this species. *BuT2* transposase is 643-aa long and contains a hATC domain, which is a highly conserved dimerization domain found in DNA transposases from the *hAT* superfamily (Essers et al. 2000). The most complete *BuT2* copy found in *D. willistoni* similarly encodes a 642-aa protein that is 90% identical to that of *D. buzzatii*. However, this copy cannot be active because it contains a nonsense mutation that results in a premature stop codon. Because the genome sequence only represents a single *D. willistoni* genome, we cannot discard the existence of active copies in other individuals or populations within this widely distributed species. As a matter of fact, the presence of many short MITE-like sequences associated to *BuT2* in *D. willistoni* genome (see later) suggests recent transpositional activity in this species.

The *hAT* superfamily is a very large and diverse group of DNA transposons and domesticated genes, as there are several examples of *hAT* superfamily elements being exapted to essential functions within the host genome (Sinzelle et al. 2009; Arensburger et al. 2011). As we mentioned before, the *hAT* superfamily consists of at least two families, *Ac* and *Buster*, based on the phylogeny of their transposases and by difference in target-site selection (Arensburger et al. 2011). Recently, Zhang et al. (2013) suggested a third group with a small number of elements that would include *Tip100* and two transposons from *B. mori* (*hAT_-4_BM*) and *R. prolixus* (*RP-hAT1*) (Zhang et al. 2013). In this work, the unrooted *hAT* transposase tree more clearly shows the presence of a third large group, comprising at least 20 sequences including *BuT2*, *Tip* transposons, *hAT_-4_BM,* and *RP-hAT1*. Thus, we propose to establish a new family of *hAT* transposons, named the *Tip* family. TSD size in this family (8 bp) seems to be similar to that of the other two, but we have not investigated the target preference that differentiates the *Ac* and *Buster* groups. Further studies of the *Tip* elements TSDs would be interesting to detect if there are similarities in the target-site selection among *Tip* elements and differences with *Ac* and *Buster* members. The *Tip* family contains sequences coming from a phylogenetically diverse array of hosts, such as *Tip100* of the common morning glory *I. purpurea* (Habu et al. 1998), *AeTip100-2* of the mosquito *Aedes aegypti* (Arensburger et al. 2011)*, hAT-12_HM* of hydra *H. magnipapillata* (Jurka 2008) and *BuT2* of *Drosophila* (Cáceres et al. 2001)*.* Here we show several other hypothetical proteins from insects are included in *Tip* clade; however, we cannot determine whether these are active TEs or domesticated genes. Up to now, *Tip* family elements are found in plants, Cnidaria, and insects from different orders Lepidoptera, Hemiptera, Hymenoptera, Coleoptera, and Diptera. Possibly, with the advancement of genome projects, other *Tip* elements will be described soon.

In insects, several *hAT* elements were characterized, such as *hobo* in *D. melanogaster* (Calvi et al. 1991), *Hermes* in *M. domestica* (Warren et al. 1994)*, Hermit* in *Lucilia cuprina* (Coates et al. 1996), *Homer* in *Bactrocera tryoni* (Pinkerton et al. 1999), *Hopper* in *Ba. dorsalis* (Handler 2003) and *Herves* in *An. gambiae* (Arensburger et al. 2005). Other *hAT* sequences were identified, by Ortiz and Loreto (2008), through in silico searches in 12 *Drosophila* genomes*.* Most of *Drosophila hAT* elements belong to *Ac* family, and recently we characterized the first *Buster* element found in *Drosophila* (Deprá et al. 2012). Here we describe the first *Tip* element in *Drosophila*.

### MITE-Like Sequences Associated to *BuT2*

Nonautonomous copies of DNA transposons are very abundant and often outnumber the canonical autonomous copies. We have found that several *Drosophila* species possess degenerated short sequences sharing similarity with the 5′ and 3′ regions of *BuT2,* including the TIRs, and might be considered MITEs although some of their characteristics were not observed. MITEs, in general, share typical structural features: 1) short elements with no coding capacity, 2) high copy number, 3) TIRs, 4) location in or near genes, and 5) AT-rich mainly in the inner region (Feschotte and Pritham 2007). This term do not represent a common origin or a taxonomic level in TE classification, although it is very useful.

The first MITE families described in *Drosophila* were *Vege* and *Mar*, both of which were discovered in *D. willistoni* (Holyoake and Kidwell 2003; Deprá et al. 2012). From there, some other TE families were described to have associated MITEs: *hobo* from *hAT* superfamily (Ortiz and Loreto 2008) *Bari* from *Tc1-Mariner* superfamily (Dias and Carareto 2011) and BuT5 from *P* superfamily (Rius et al. 2013). Here we show *BuT2* also has associated MITE sequences. *BuT2* MITEs, as suggested for other MITE elements (Jiang et al. 2003, 2004; Zhang et al. 2004; Ortiz and Loreto 2008; Deprá et al. 2012), seem to originate by internal deletion of the autonomous element, and during the host species evolution, these MITEs may have originated independently in ancestor and present species. We were unable to analyze the number of copies or conservation of TIRs and TSDs in species other than *D. willistoni*; thus, we do not know whether the *BuT2* MITEs spread successfully throughout other genomes.

The number of MITE copies found in *D. willistoni* (24 copies) is not as high as that of most of the plants and mosquito MITE families, but there are several families exhibiting more modest copy numbers (Jiang et al. 2003; Quesneville et al. 2006; Grzebelus et al. 2009; Yang et al. 2009; Xu et al. 2010). The *D. willistoni BuT2* MITEs present conserved TIRs and TSDs and are grouped together within the same clade of the phylogenetic tree (fig. 3*A*), suggesting recent mobilization and amplification. The mechanisms of MITE amplification remain poorly understood; but for *BuT2* MITES apparently, it could not be explained by duplications. In general, TSD sequences of a copy are identical, but different from those from the other copies, indicating the copies are amplified by a transposition rather than a duplication mechanism, also implicating the presence of an active transposase. Because of the conserved elements ends, that are required for transposition, the most likely hypothesis at this moment is that these MITES are mobilized by the transposase encoded by an active *BuT2* copy. Another less likely hypothesis is that *BuT2* MITEs can be mobilized by other *hAT* active element. Cross-mobilization is highly associated with the amplification of MITE families (Jiang et al. 2003; Torres et al. 2006; Yang et al. 2009). Within the *hAT* superfamily, cross-mobilization has been reported for the *hobo* element, which is able to mobilize the *hermes* transposons (Sundararajan et al. 1999). However, both elements belong to the same *hAT* family, the *Ac* (see fig. 2) that probably helps on this process.

### *BuT2* Is Involved in Multiple Events of HT

**Fig. 5.—:**
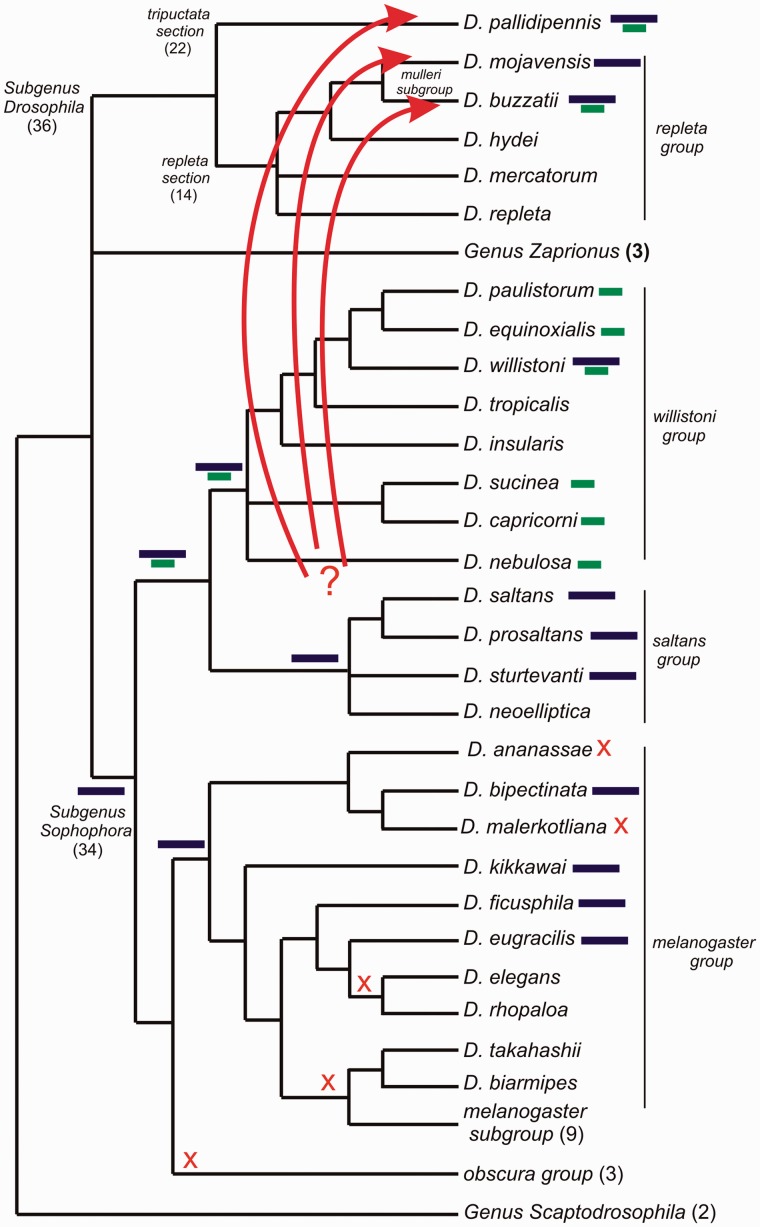
Scheme of phylogenetic relationships of different Drosophilidae species groups employed in this study based on several works (Lewis et al. 2005; Robe et al. 2005; Kopp 2006; Robe, Cordeiro, et al. 2010; Robe, Loreto, et al. 2010; Oliveira et al. 2012). Numerous species that do not have *BuT2* were omitted and only the number of species tested is shown. Blue and green bars near species represent the distribution of *BuT2* complete copies and MITEs, respectively. The bars at nodes point out the potential presence of those sequences in the main ancestors. Orange crosses represent possible lost events of *BuT2,* and red arrows indicate probable cases of HT from some species of *willistoni* or *saltans* groups to *D. pallidipennis*, *D. mojavensis,* and *D. buzzatii*.

To reconstruct the *BuT2* evolutionary history in the genus *Drosophila*, we analyzed its interspecific distribution using a combination of bioinformatic and experimental approaches. The results are summarized in figure 5. Three different kinds of evidence are usually considered to indicate HT of TEs: 1) Patchy distribution of a TE across a group of species; 2) Incongruence between host and TE phylogenies; and 3) High sequence similarity between TEs of distantly related species (Silva et al. 2004). We found *BuT2* homologous sequences in species from five *Drosophila* groups with patchy distribution, incongruities between *BuT2* and host phylogenies, and high similarity between copies belonging to distantly related species. Therefore, we can conclude that *BuT2* has been horizontally transferred between some *Drosophila* species.

Despite the low support of some branches of the *BuT2* phylogenies, we can observe a patchy distribution of *BuT2* and some well-supported incongruities when compared with the host species phylogenetic relationships (fig. 3*A* and *B*). In the *tripunctata* section of subgenus *Drosophila*, *BuT2* was found in only one (*D. pallidipennis*) among 22 species tested (fig. 5). In the *repleta* section, *BuT2* is present in two closely related species, *D. buzzatii*, where it was originally found, and *D. mojavensis*. Three other species from the same group (*D. repleta, D. mercatorum,* and *D. hydei*) do not contain *BuT2* sequences (fig. 5). This distribution is not consistent with VT. In the subgenus *Sophophora,* we observed a widely distribution of *BuT2* in the species from the sister groups *saltans* and *willistoni*. This broadly distribution is consistent with the presence of *BuT2* element in the ancestor of these two groups (fig. 5). We can also find *BuT2* sequences in some species of *melanogaster* group, however, with a scattered distribution suggesting HT events and/or stochastic loss.

Divergences in TE sequences lower than the divergence between nuclear genes of their respective host species are also indicative of HT (Silva et al. 2004; Wallau et al. 2012). Using as control two genes, *Adh* and *Amd*, we have found *BuT2* divergence to be significantly lower than expected when comparing *D. pallidipennis* with *D. willistoni, D. buzzatii* and *D. mojavensis* with all *willistoni* and *saltans* species, and also *D. buzzatii* with *D. mojavensis.*

Taking together all results, we can postulate a possible scenario (fig. 5): a *BuT2-like* copy was present in the ancestor of subgenus *Sophophora,* and during speciation process, it was completely lost independently in several species of *melanogaster* and *obscura* groups, although few species of *melanogaster* group still have remnants of this transposon. This *BuT2-like* element, nonetheless, was apparently maintained active in species of *willistoni* and *saltans* group while was vertically transmitted during species evolution. To explain the presence of *BuT2* in some species of subgenus *Drosophila,* we need to propose three HT events: First, from a species of *saltans* or *willistoni* subgroups to *D. pallidipennis*; and more recently, another two cases also from a species of *saltans* or *willistoni* subgroups to *D. mojavensis* and *D. buzzatii* independently. Alternatively, one of the HT events could have occurred between these two species. A more parsimonious explanation would be an HT event to the ancestor of these two species; however, the lower divergence found for *BuT2* is inconsistent with this hypothesis, unless *BuT2* is under a higher selective constraint than *Adh* gene in these species, which is unlikely.

HT events have been proposed as a key step in the TE lifecycle, allowing these sequences to escape extinction before inactivation into the host genome (Le Rouzic and Capy 2006; Venner et al. 2009; Hua-Van et al. 2011). Once in the new genome, the horizontally transferred TE can generate mutations in the same way as those vertically transmitted with detrimental consequences. However, HT can be an important mechanism of genetic innovation, because a newly arrived TE consists in new regulatory and coding regions available to be co-opted by the host genome (Thomas et al. 2010). Also, TEs can facilitate the transfer of additional genetic material and play an important role in the responsive capacity of their hosts to environmental changes (Frost et al. 2005; Casacuberta and González 2013).

The mechanisms for HTs remain obscure, although these transfer events require the occurrence of common premises, such as geographical, temporal, and ecological overlap between donor and recipient species. The species involved in our work are widespread in Neotropics and share some ecological resources (Schmitz et al. 2007); therefore, the conditions for the HT of the *BuT2* element are present.

Our work revealed *But2* has a multifaceted evolution and life cycle, becoming an important example to investigate the behavior of *hAT* TEs in the eukaryotes, effects of HT in the receptor genomes, the implications generated by the coexistence of complete copies and MITEs, and the dynamics of MITE amplification in the host genomes.

## Supplementary Material

Supplementary figure S1 and tables S1–S6 are available at *Genome Biology and Evolution* online (http://www.gbe.oxfordjournals.org/).

Supplementary Data
