# Supplementary material for: BuT2 Is a Member of the Third Major Group of hAT Transposons and Is Involved in Horizontal Transfer Events in the Genus Drosophila
Source: Genome Biol Evol. 2014 Jan 22;6(2):352–65. doi: 10.1093/gbe/evu017 (PMC3942097; doi:10.1093/gbe/evu017)
Supplement: Supplementary Data [file supp_6_2_352__index.html]

BuT2 Is a Member of the Third Major Group of hAT Transposons and Is Involved in Horizontal Transfer Events in the Genus Drosophila — Supplementary Data 

# *BuT2* Is a Member of the Third Major Group of *hAT* Transposons and Is Involved in Horizontal Transfer Events in the Genus *Drosophila*

## Supplementary Data

files

**Files in this Data Supplement:**

- Supplementary Data - pdf file
- Supplementary Data - pdf file
- Supplementary Data - pdf file
- Supplementary Data - pdf file
- Supplementary Data - pdf file
- Supplementary Data - pdf file
- Supplementary Data - pdf file
