# Supplementary material for: BuT2 Is a Member of the Third Major Group of hAT Transposons and Is Involved in Horizontal Transfer Events in the Genus Drosophila
Source: Genome Biol Evol. 2014 Jan 22;6(2):352–65. doi: 10.1093/gbe/evu017 (PMC3942097; doi:10.1093/gbe/evu017)
Supplement: Supplementary Data [file supp_evu017_Supplementary_Figure_S1.pdf]

Supplementary Figure S1

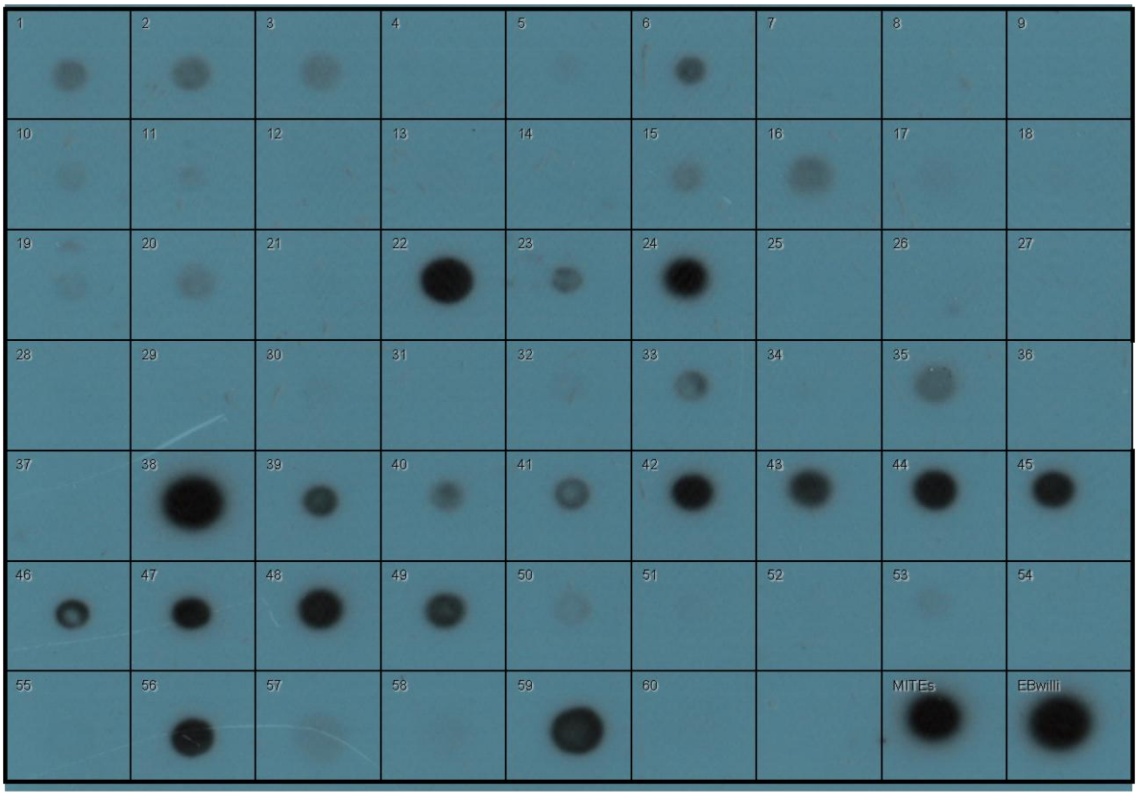

Supplementary Figure S1: Dot blot screening for presence of *But2*. The species tested are the followed: *1 – D. ornatifrons; 2 – D. subbadia; 3 – D. guaru; 4 – D. griseolineata; 5 – D. maculifrons; 6 –D. nappae; 7 – D. paramediostriata; 8 – D. tripunctata; 9 – D. mediodiffusa; 10 – D. mediopictoides; 11 – D. cardinoides; 12 – D. neocardini; 13 – D. polymorpha; 14 – D. procardinoides; 15 – D. arawakana; 16 – D. ornatipennis; 17 – D. immigrans; 18 – D. funebris; 19 – D. gasici; 20 – D. brncici; 21 – D. gaucha; 22 – D. buzzatii; 23 – D. mercatorum; 24 – D. incompta; 25 – D. virilis; 26 – D. robusta; 27 – D. melanogaster; 28 – D. mauritiana; 29 – D. teissieri; 30 – D. santomea; 31 – D. erecta; 32 – D. yakuba; 33 – D. kikkawai; 34 – D. ananassae; 35 – D. malerkotliana; 36 – D. orena; 37 – D. pseudoobscura; 38 – D. prosaltans; 39 – D. saltans; 40 – D. neoelliptica; 41 – D. sturtevanti; 42 – D. sucinea; 43 – D. nebulosa; 44 – D. paulistorum; 45 – D. willistoni; 46 – D. equinoxialis; 47 – D. tropicalis; 48 – D. insularis; 49 – D. capricorni; 50 – D. busckii; 51 – Z. indianus; 52 – Z. tuberculatus; 53 – S. latifasciaeformis; 54 – S. lebanonensis; 55 – D. crocina; 56 – D. pallidipennis; 57 – D. pavani; 58 – D. hydei; 59 – D. mojavensis; 60 – D. simulans*; Bf1_willi – *D. willistoni* clone (MITE); Bf2_willi – *D. willistoni* clone (probe).
