# Supplementary material for: BuT2 Is a Member of the Third Major Group of hAT Transposons and Is Involved in Horizontal Transfer Events in the Genus Drosophila
Source: Genome Biol Evol. 2014 Jan 22;6(2):352–65. doi: 10.1093/gbe/evu017 (PMC3942097; doi:10.1093/gbe/evu017)
Supplement: Supplementary Data [file supp_evu017_Supplementary_Table_S1.pdf]

Supplementary Table S1: Drosophilidae stocks used in this work and their origin.

Genus	Subgenus	Group	Species	Stock origin
Drosophila	*Drosophila*	*guarani*	*D. ornatifrons*	Porto Alegre, RS, Brazil
			D. subbadia	El Narango, Mexico
			D. guaru	Amalia, SP, Brazil
		grimshawi	*D. grimshawi*	Unknown
		guaramuru	*D. griseolineata*	PortoAlegre, RS, Brazil
			D. maculifrons	Santa Maria, RS, Brazil
		tripunctata	*D. nappae*	Itapuã, RS, Brazil
			D. paraguayensis	Porto Alegre, RS, Brazil
			D. crocina	Unknown, Brazil
			D. paramediostriata	Porto Alegre, RS, Brazil
			D. tripunctata	Iowa River, Iowa, USA
			D. mediodiffusa	Maricao, Porto Rico
			D. mediopictoides	Boquete, Panama
		cardini	*D. cardini*	Itaqui, RS, Brazil
			D. cardinoides	Porto Alegre, RS, Brazil
			D. neocardini	Florianópolis, SC, Brazil
			D. polymorpha	Porto Alegre, RS, Brazil
			D. procardinoides	Coroico, Bolivia
			D. arawakana	St Kitts, Caribe
		pallidipennis	*D. pallidipennis*	Joinville, SC, Brazil
		calloptera	*D. ornatipennis*	Guadalupe Island, Caribe
		immigrans	*D. immigrans*	Porto Alegre, RS, Brasil
		funebris	*D. funebris*	Mexico City, Mexico
		mesophragmatica	*D. gasici*	Arica, Chile
			D. brncici	Bogota, Colombia
			D. gaucha	Campos do Jordão, SP, Brazil
			D. pavani	La Florida, Chile
		repleta	*D. hydei*	Porto Alegre, RS, Brazil
			D. mojavensis	Stock Center
			D. buzzatii	Unknown
			D. mercatorum	Porto Alegre, RS, Brazil
			D. repleta	Stock Center
		canalinea	*D. canalinea*	
		flavopilosa	*D. cestri*	Santa Maria, RS, Brazil
			D. incompta	Santa Maria, RS, Brazil
		virilis	*D. virilis*	Unknown, Brazil
		robusta	*D. robusta*	Unknown
	Sophophora	*melanogaster*	*D. melanogaster*	Harwich, Bowling Green, EUA
			D. simulans	Florianópolis, SC, Brazil
			D. sechellia	Sheycheles
			D. mauritiana	Tucson Stock Center, EUA
			D. teissieri	Africa

			D. santomea	Stock Center
			D. erecta	Stock Center
			D. yakuba	Stock Center
			D. kikkawai	Porto Alegre, RS, Brazil
			D. ananassae	Florianópolis, SC, Brazil
			D. malerkotliana	Florianópolis, SC, Brazil
			D. orena	Stock Center
		obscura	*D. pseudoobscura*	Unknown
		saltans	*D. prosaltans*	Unknown, Brazil
			D. saltans	Unknown, Brazil
			D. neoelliptica	Unknown, Brazil
			D. sturtevanti	Florianópolis, SC, Brazil
		willistoni	*D. sucinea*	Porto Alegre, RS, Brazil
			D. nebulosa	Porto Alegre, RS, Brazil
			D. paulistorum	Florianópolis, SC, Brazil
			D. willistoni	Porto Alegre, RS, Brazil
			D. equinoxialis	Tefé, AM, Brazil
			D. insularis	St Kitts, Lesser Antilles
			D. tropicalis	San Salvador, El Salvador
			D. capricorni	Florianópolis, SC, Brazil
	Dorsilopha		*D. busckii*	Uruguai
Zaprionus			*Z. indianus*	Porto Alegre, RS, Brazil
			Z. tuberculatus	Unknown
			Z. sepsoide	Unknown
Scaptodrosophila			*S. latifasciaeformis*	São José do Rio Preto, SP, Brazil
			S. lebanonensis	Stock Center

States: (RS) Rio Grande do Sul; (SC) Santa Catarina; (SP) São Paulo; (AM) Amazônia.
