# Supplementary material for: BuT2 Is a Member of the Third Major Group of hAT Transposons and Is Involved in Horizontal Transfer Events in the Genus Drosophila
Source: Genome Biol Evol. 2014 Jan 22;6(2):352–65. doi: 10.1093/gbe/evu017 (PMC3942097; doi:10.1093/gbe/evu017)
Supplement: Supplementary Data [file supp_evu017_Supplementary_Table_S2.pdf]

Supplementary Table S2: List of protein sequences used in the article. They were collected from several databases and two manuscripts.

Element	Source/Accession number	Species
	**Genbank**	
Ac-like	AAC46515	*Caenorhabditis elegans*
Ac	CAA29005	*Zea mays*
AeBuster1	ABF20543	*Aedes aegypti*
AeBuster2	ABF20544	*Aedes aegypti*
AmBuster1	EFB22616	*Ailuropoda melanoleuca*
AmBuster2	EFB25016	*Ailuropoda melanoleuca*
AmBuster3	EFB20710	*Ailuropoda melanoleuca*
AmBuster4	EFB22020	*Ailuropoda melanoleuca*
BtBuster1	ABF22695	*Bos taurus*
BtBuster2	ABF22700	*Bos taurus*
BtBuster3	ABF22697	*Bos taurus*
CfBuster1	ABF22696	*Canis lupus familiaris*
CfBuster2	ABF22701	*Canis lupus familiaris*
CfBuster3	XP_854762	*Canis lupus familiaris*
CfBuster4	XP_545451	*Canis lupus familiaris*
CsBuster	ABF20548	*Ciona savignyi*
Daysleeper	CAB68118	*Arabidopsis thaliana*
DrBuster1	ABF20549	*Danio rerio*
DrBuster2	ABF20550	*Danio rerio*
EcBuster1	XP_001504971	*Equus caballus*
EcBuster3	XP_001503499	*Equus caballus*
EcBuster4	XP_001504928	*Equus caballus*
Hermes	AAC37217	*Musca domestica*
Herves	AAS21248	*Anopheles gambiae*
hobo	A39652	*Drosophila melanogaster*
Homer	AAD03082	*Bactrocera tryoni*
hopper-we	AAL93203	*Bactrocera tryoni*
HsBuster1	AAF18454	*Homo sapiens*
HsBuster2	ABF22698	*Homo sapiens*
HsBuster3	NP_071373	*Homo sapiens*
HsBuster4	AAS01734	*Homo sapiens*
IpTip100	BAA36225	*Ipomoea purpurea*
MamBuster2	XP_001108973	*Macaca mulatta*
MamBuster3	XP_001084430	*Macaca mulatta*
MamBuster4	XP_001101327	*Macaca mulatta*
MmBuster2	AAF18453	*Mus musculus*
PtBuster2	ABF22699	*Pan troglodytes*
PtBuster3	XP_001142453	*Pan troglodytes*
PtBuster4	XP_527300	*Pan troglodytes*
Restless	CAA93759	*Tolypocladium inflatum*
RnBuster2	NP_001102151	*Rattus norvegicus*
SpBuster1	ABF20546	*Strongylocentrotus purpuratus*

SpBuster2	ABF20547	*Strongylocentrotus purpuratus*
SsBuster4	XP_001929194	*Sus scrofa*
Tam3	CAA38906	*Antirrhinum majus*
TcBuster	ABF20545	*Tribolium castaneum*
Tol2	BAA87039	*Oryzias latipes*
tramp	CAA76545	*Homo sapiens*
XtBuster	ABF20551	*Xenopus Silurana tropicalis*
	**ENSEMBL**	
PtBuster1	ENSPTRG00000003364	*Pan troglodytes*
	**REPBASE**	
Ac-like2 (hAT-7_DR)		*Danio rerio*
Ac-like1 (hAT-6_DR)		*Danio rerio*
hAT5_DR		*Danio rerio*
MlBuster1 (hAT-4_ML)		*Myotis lucifugus*
Myotis-hAT1		*Myotis lucifugus*
SPIN_Et		*Echinops telfairi*
SPIN_Ml		*Myotis lucifugus*
SPIN-Og		*Otolemur garnettii*
hAT-29_HM		*Hydra magnipapillata*
hAT6-1_NVp		*Nasonia vitripennis*
hAT-46_HM		*Hydra magnipapillata*
hAT-12_HM		*Hydra magnipapillata*
	**TEFam**	
AeHermes1	TF0013337	*Aedes aegypti*
AeBuster3	TF001186	*Aedes aegypti*
AeBuster4	TF001187	*Aedes aegypti*
AeBuster5	TF001188	*Aedes aegypti*
AeBuster7	TF001336	*Aedes aegypti*
AeHermes2	TF0013338	*Aedes aegypti*
AeTip100-2	TF000910	*Aedes aegypti*
	**BLASTp/Genbank**	
CfloZFN-protein	EFN60310	*Camponotus floridanus*
TcasGA2_TC012970	EFA13101	*Tribolium castaneum*
TcasGA2_TC016136	EFA12008	*Tribolium castaneum*
LOC100575841	XP_003241537	*Acyrthosiphon pisum*
LOC100572638	XP_003240128.1	*Acyrthosiphon pisum*
LOC100572555	XP_003240127.1	*Acyrthosiphon pisum*
TcasGA2_TC001388	EFA11723.1	*Tribolium castaneum*
LOC100573118	XP_003241183.1	*Acyrthosiphon pisum*
LOC100574776	XP_003241529.1	*Acyrthosiphon pisum*
TcasGA2_TC005046	EFA11959.1	*Tribolium castaneum*
LOC100680020	XP_003424282.1	*Nasonia vitripennis*

	**Others**	
RP-hAT1	Zhang et al (2013)	*Rhodnius prolixus*
hAT-4_BM	Zhang et al (2013)	*Bombyx mori*
Mar	Deprá et al (2012)	*Drosophila willistoni*
harrow	Mota et al (2010)	*Drosophila willistoni*
