# Supplementary material for: BuT2 Is a Member of the Third Major Group of hAT Transposons and Is Involved in Horizontal Transfer Events in the Genus Drosophila
Source: Genome Biol Evol. 2014 Jan 22;6(2):352–65. doi: 10.1093/gbe/evu017 (PMC3942097; doi:10.1093/gbe/evu017)
Supplement: Supplementary Data [file supp_evu017_Supplementary_Table_S3.pdf]

Supplementary Table S3: Genbank accession number or scaffold position of *Amd* and *Adh* genes sequences

	Amd	***Adh***
D. willistoni	FJ664508	U95264
D. sucinea	FJ664510	
D. equinoxialis	FJ664506	
D. tropicalis	FJ664504	
D. paulistorum	FJ664496	AB026529
D. insularis	FJ664507	
D. fumipennis	FJ664509	
D. pallidipennis	EU447332	
D. bipectinata	gi|459198956:1537766-1538592	gi|459198968:1052699-1053000
D. kikkawai	gi|459202748:729693-729918	gi|459202934:988575-988932
D. ficusphila	gi|459201402:430017-430242	gi|459201661:142836-14319
D. eugracilis	gi|459206075:1441836-1442046	gi|459206080:1182489-1182852
D. mojavensis	scaffold_6500:6793410-6793582	XM_002002894
D. prosaltans		AF045119
D. sturtevanti		AB026535
D. saltans		AF045113
