# Supplementary material for: BuT2 Is a Member of the Third Major Group of hAT Transposons and Is Involved in Horizontal Transfer Events in the Genus Drosophila
Source: Genome Biol Evol. 2014 Jan 22;6(2):352–65. doi: 10.1093/gbe/evu017 (PMC3942097; doi:10.1093/gbe/evu017)
Supplement: Supplementary Data [file supp_evu017_Supplementary_Table_S4.pdf]

Supplementary Table S4: *But2* sequences identified in the *Drosophila* genomes with the nomenclature used in this work, length and the location in the scaffolds.

Species	Scaffold position	Name	Length (bp)
D. ficusphila	scf7180000452383: 782-1011	scf1_Dfis	229
	scf7180000449450: 23043 - 23265	scf2_ Dfis	222
	scf7180000453926: 4228-4504	scf3_ Dfis	276
D. eugracilis	scf7180000409744:10071-10445	scf1_Deug	374
	scf7180000409744: 8253 - 9091	scf2_ Deug	836
	scf7180000409744:9531-9812	scf3_ Deug	281
D. kikkawai	scf7180000301804:11122-11466	scf1_Dkik	344
	scf7180000301804:11727-12561	scf2_ Dkik	834
	scf7180000301988:3029-3194	scf3_ Dkik	165
D. bipectinata	scf7180000391773: 3206 - 3418	scf1_Dbip	212
D. mojavensis	scaffold_3367: 3535-7555	scf1_Dmoj	4017
	scaffold_6500: 28308611-28313538	scf2_ Dmoj	4928
	scaffold_6498: 3233207-3234334	scf3_ Dmoj	1128
D. willistoni*	scf2_1100000004958:2664879-2668034	scf1_Dwil	3156
	scf2_1100000004967:1340158-1346251	scf2_ Dwil	6092

* For these species, information of several hits representing *But2* MITE copies were omitted from this table and are available on Supplementary Table S4
