# Supplementary material for: BuT2 Is a Member of the Third Major Group of hAT Transposons and Is Involved in Horizontal Transfer Events in the Genus Drosophila
Source: Genome Biol Evol. 2014 Jan 22;6(2):352–65. doi: 10.1093/gbe/evu017 (PMC3942097; doi:10.1093/gbe/evu017)
Supplement: Supplementary Data [file supp_evu017_Supplementary_Table_S5.pdf]

Supplementary Table S5: Short sequences related to *But2* identified in the *D. willistoni* genome with the nomenclature used in this work, the scaffolds positions, their respective TSD and TIR sequences and total length.

Name	Scaffold position	TSD	TIR	Length (bp)
**Scf3_Dwil**	scf2_1100000004962:321475-322145	GCGAGAGC	CAGTGCTGCCAA	671
**Scf4_Dwil**	scf2_1100000004953:192719-193643	TTT(C/T)GAAG	CAGTGCTGCCAA	925
**Scf5_Dwil**	scf2_1100000004953:1127087-1128013	GTCGCTAC	CAGTGCTGCCAA	927
**Scf6_Dwil**	scf2_1100000004949:1398397-1397471	CCGGCAGC	CAGTGCTGCCAA	927
**Scf7_Dwil**	scf2_1100000004954:3708901-3709527	ACTAGAAG	CAGTGCTGCCAA	627
**Scf8_Dwil**	scf2_1100000004943:8134656-8135187	ACTT(T/C)GAG	CAGTGCTGCCAA	532
**Scf9_Dwil**	scf2_1100000004943:1484483-1485186	CCT(T/C)TGCG	CAGTGCTGCCAA	704
**Scf10_Dwil**	scf2_1100000004962:232665-233445	(G/T)ACGAAA(C/G)	CAGTGCTGCCAA	781
**Scf11_Dwil**	scf2_1100000004511:9081994-9081209	AGTGGAAG	CAGTGCTGCCAA	786
**Scf12_Dwil**	scf2_1100000004839:28485-29274	CCGCGACG	CAGTGCTGCCAA	790
**Scf13_Dwil**	scf2_1100000004830:149935-150707	CTCACCG(G/C)	C(A/G)GTGCTGCCAA	773
**Scf14_Dwil**	scf2_1100000004954:3908919-3909661	TCTAGTAG	CAGTGCTGCCAA	743
**Scf15_Dwil**	scf2_1100000004943:5612842-5613666	Not conserved	CAGTGCTGC(C/A)AA	825
**Scf16_Dwil**	scf2_1100000004949:2237607-2238399	C(T)GCAGATC	CAGTGCTGCCAA	793
**Scf17_Dwil**	scf2_1100000004513:4294147-4294844	Not conserved	(C/T)AGTG(C/A)TGCCAA	698
**Scf18_Dwil**	scf2_1100000004542:147368-146573	CATACAAC	CAGTG(C/T)TGCCAA	796
**Scf19_Dwil**	scf2_1100000004585:8660413-8659622	TTCACAGC	C(A/T)GTGCTGCCAA	792
**Scf20_Dwil**	scf2_1100000004943:13787775-13787114	GTTGGAA(G/T)	CAGTGCTGCCAA	662
**Scf21_Dwil**	scf2_1100000004954:4086836-4086268	CATGTCTG	CAGTGCTGCCAA	646
**Scf22_Dwil**	scf2_1100000004943:9179935-9180716	CGCGCACT	CAGTGCTGCCAA	782
**Scf23_Dwil**	scf2_1100000004943:6218169-6218755	GCGACATG	CAGTGC(T/C)GCCAA	587
**Scf24_Dwil**	scf2_1100000004953:522711-523472	ATGACAGC	CAGTGCTGCCAA	762
**Scaf25_Dwil**	scf2_1100000004521:10176460-10175847	Not conserved	CAGTGCTGCCAA	614
**Scf26_Dwil**	scf2_1100000004902:5686245-5686846	Not conserved	CAGTGCTGCCAA	602
