# Supplementary material for: BuT2 Is a Member of the Third Major Group of hAT Transposons and Is Involved in Horizontal Transfer Events in the Genus Drosophila
Source: Genome Biol Evol. 2014 Jan 22;6(2):352–65. doi: 10.1093/gbe/evu017 (PMC3942097; doi:10.1093/gbe/evu017)
Supplement: Supplementary Data [file supp_evu017_Supplementary_Table_S6.pdf]

Supplementary Table S6: GenBank accession number of clones obtained in our work with the nomenclature used.

Species	Name	Accession number
Drosophila equinoxialis clone 1	Bf1_Dequ1	KF669618
Drosophila buzzatii clone 4	Bf1_Dbuz4	KF669619
Drosophila pallidipennis clone 2	Bf1_Dpap2	KF669620
Drosophila pallidipennis clone 1	Bf1_Dpap1	KF669621
Drosophila sucinea clone 4	Bf1_Dsuc4	KF669622
Drosophila sucinea clone 1	Bf1_Dsuc1	KF669623
Drosophila sucinea clone 2	Bf1_Dsuc2	KF669624
Drosophila sucinea clone 3	Bf1_Dsuc3	KF669625
Drosophila capricorni clone 1	Bf1_Dcap1	KF669626
Drosophila buzzatii clone 3	Bf1_Dbuz3	KF669627
Drosophila buzzatii clone 1	Bf1_Dbuz1	KF669628
Drosophila buzzatii clone 2	Bf1_Dbuz2	KF669629
Drosophila buzzatii clone 5	Bf1_Dbuz5	KF669630
Drosophila buzzatii clone 6	Bf1_Dbuz6	KF669631
Drosophila nebulosa clone 1	Bf1_Dneb1	KF669632
Drosophila nebulosa clone 2	Bf1_Dneb2	KF669633
Drosophila paulistorum clone 1	Bf1_Dpau1	KF669634
Drosophila paulistorum clone 2	Bf1_Dpau2	KF669635
Drosophila pallidipennis clone 3	Bf2_Dpap3	KF669636
Drosophila buzzatii clone 7	Bf2_Dbuz7	KF669637
Drosophila sturtevanti clone 1	Bf2_Dstu1	KF669638
Drosophila prosaltans clone 1	Bf2_Dpro1	KF669639
Drosophila saltans clone 1	Bf2_Dsal1	KF669640
Drosophila willistoni clone 1	Bf2_Dwil1	KF669641
